# 3D-printed external fixation guide combined with video-assisted thoracoscopic surgery for the treatment of flail chest: a technical report and case series

**DOI:** 10.3389/fsurg.2023.1272628

**Published:** 2023-09-27

**Authors:** Meng Hu, Maolin Sun, Chuanen Bao, Junlong Luo, Longcai Zhuo, Ming Guo

**Affiliations:** Department of Cardiothoracic Surgery, Xiamen University Affiliated Chenggong Hospital (Army 73rd Group Military Hospital), Xiamen, China

**Keywords:** 3D printing, thoracoscopy, external fixation, flail chest, minimally invasive, clinical efficacy

## Abstract

**Background:**

Flail chest is a common and serious traumatic condition in thoracic surgery. The treatment of flail chest often includes open reduction and internal fixation, which is relatively traumatic, complicated, and expensive. As three-dimensional (3D) printing technology is widely used in the clinical field, the application of 3D-printed products to chest trauma will become a new treatment option. To date, the use of 3D-printed external fixation guides for flail chests has not been reported. Thus, we aimed to assess the short-term efficacy of a new technology that treated flail chests with an individualized 3D-printed external fixation guide combined with video-assisted thoracoscopic surgery (VATS).

**Patients and methods:**

A retrospective analysis was performed on patients with flail chest treated with this new technique at our center from January 2020 to December 2022. The following parameters were included: operative time, thoracic tube extraction time, intensive care unit time, thoracic volume recovery rate, visual analog scale score 1 month postoperatively, and postoperative complication rate. All patients were followed up for at least 3 months.

**Results:**

Five patients (mean age: 45.7 years) were enrolled; they successfully underwent surgery without chest wall deformity and quickly returned to daily life. The average number of rib fractures was 8.4; all patients had lung contusion, hemopneumothorax, and anomalous respiration. The abnormal breathing of all patients was completely corrected on postoperative day 1, and the chest wall was stable. One case experienced mild loosening of the 3D-printed guide postoperatively; however, the overall stability was not affected. The other four cases did not experience such loosening because we replaced the ordinary silk wire with a steel wire. All cases were discharged from the hospital 2 weeks postoperatively and returned to normal life 1 month after the removal of the 3D-printed guide on average. Only one case developed a superficial wound infection postoperatively, and no perioperative death occurred.

**Conclusions:**

The 3D-printed external fixation guide combined with video-assisted thoracoscopic surgery is a novel technique in the treatment of flail chest and is safe, effective, feasible, and minimally invasive, with satisfactory clinical efficacy.

## 1. Introduction

Flail chest is a severe external thoracic trauma that leads to at least two or more rib fractures and abnormal breathing and has an impact on normal ventilation, with a high mortality rate (1, 2). Severe pain can worsen breathing difficulties. Flail chest is the second most common cause of death from chest trauma after chest vascular injury (3). Open reduction and internal fixation (ORIF) of ribs is often used to resolve the instability of the chest wall in patients with flail chest (4, 5). ORIF is beneficial in reducing complications associated with flail chest and improving the long-term quality of life and functions of patients; it has satisfactory clinical efficacy (6, 7). With the rapid development of three-dimensional (3D) printing technology, the application of this technology in the treatment of flail chests will become a new treatment option (8–11).

We herein used a new material to design an external fixation guide with computer-aided design (CAD) and 3D printing technology. The floating ribs were fixed on the guide combined with video-assisted thoracoscopic surgery (VATS). This is the first clinical report with a series of treated patients in whom abnormal breathing was corrected, pain symptoms were relieved, and thoracic volume was restored; thus, this technique has great potential for clinical application as it can achieve satisfactory clinical efficacy.

## 2. Materials and surgical technique

### 2.1. Patient selection

Inclusion criteria: (1) Multiple rib fractures after chest trauma, diagnosed with callus chest with abnormal breathing; (2) No serious intrathoracic organ injury; (3) No multiple injuries or combined injuries; (4) No surgical contraindications; (5) Be willing to follow up. Exclusion criteria: (1) Patients with critical complications such as progressive hemothorax; (2) Do not accept this kind of new surgery method; (3) Refused to follow-up. In this study, a 3D-printed external fixation guide combined with VATS was performed by a senior surgeon with rich clinical experience. This study was approved by the Medical Ethics Committee of Xiamen University Affiliated Chenggong Hospital, and all patients signed informed consent (No. 73JYY2023110463).

### 2.2. Key points of 3D-printed external fixation guide

**Figure 1:**
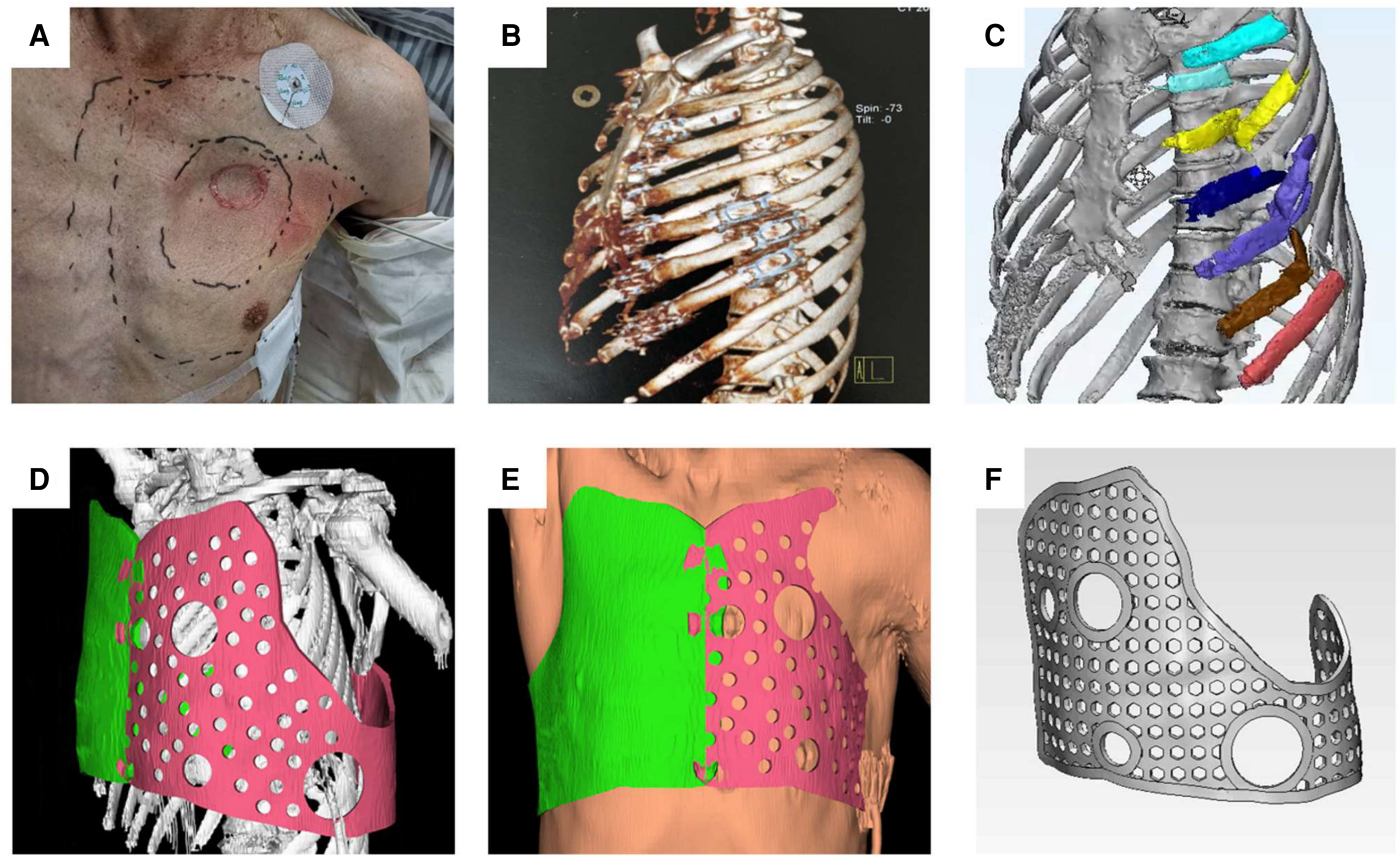
The range of rib fractures, the location of paradoxical breathing, and the approximate position of the 3D-printed guide were determined. (**A**) According to the patient's CT data, three-dimensional reconstruction was performed to determine the specific number and location of rib fractures. (**B**) DICOM data derived from CT were imported into MIMICS software, modeling was performed, and rib fracture regions were marked in color. (**C**) According to the morphological data of the healthy side of the thorax, the external fixation guide was designed. (**D**) Soft tissue was added to the model and the size of the external fixation guide was adjusted to simulate a tight fit with the chest wall. (**E**) Several holes were designed in the guide for positioning and fixing (**F**).

First, the boundary to be fixed was marked based on the extent of the patients' flail chest injury. The number of rib fractures and the state of injury can be confirmed in this model. We performed 3D reconstruction of the thoracic morphology using patients' computed tomography (CT) data (DICOM format). The data was then imported into Mimics software, and the rib fracture area was marked with other colors. The shape of the external fixation guide was designed according to the condition of the healthy chest wall. The aim was to completely enclose the area with the rib fracture (colored area). The guide has several holes through which the steel wire can be inserted during the surgery to play the role of external fixation. Furthermore, four larger holes were indwelled on the surface of the guide plate for thoracoscopic access and surgical instrument insertion. To ensure a good surgical field of vision and sufficient operating space, the guide plate fixed in the chest wall had a hole in four directions (upper, lower, front, and rear). The shape of the chest wall on both sides after reconstruction was compared again using Mimics software to ensure that the shape of the chest wall on the affected side was symmetrical to that of the healthy side, the chest volume was effectively restored, and the guide was effectively fixed to the damaged side. Lastly, the final shape of the guide was obtained by removing the chest wall image (Figure 1).

### 2.3. Key steps in the procedure

**Figure 2:**
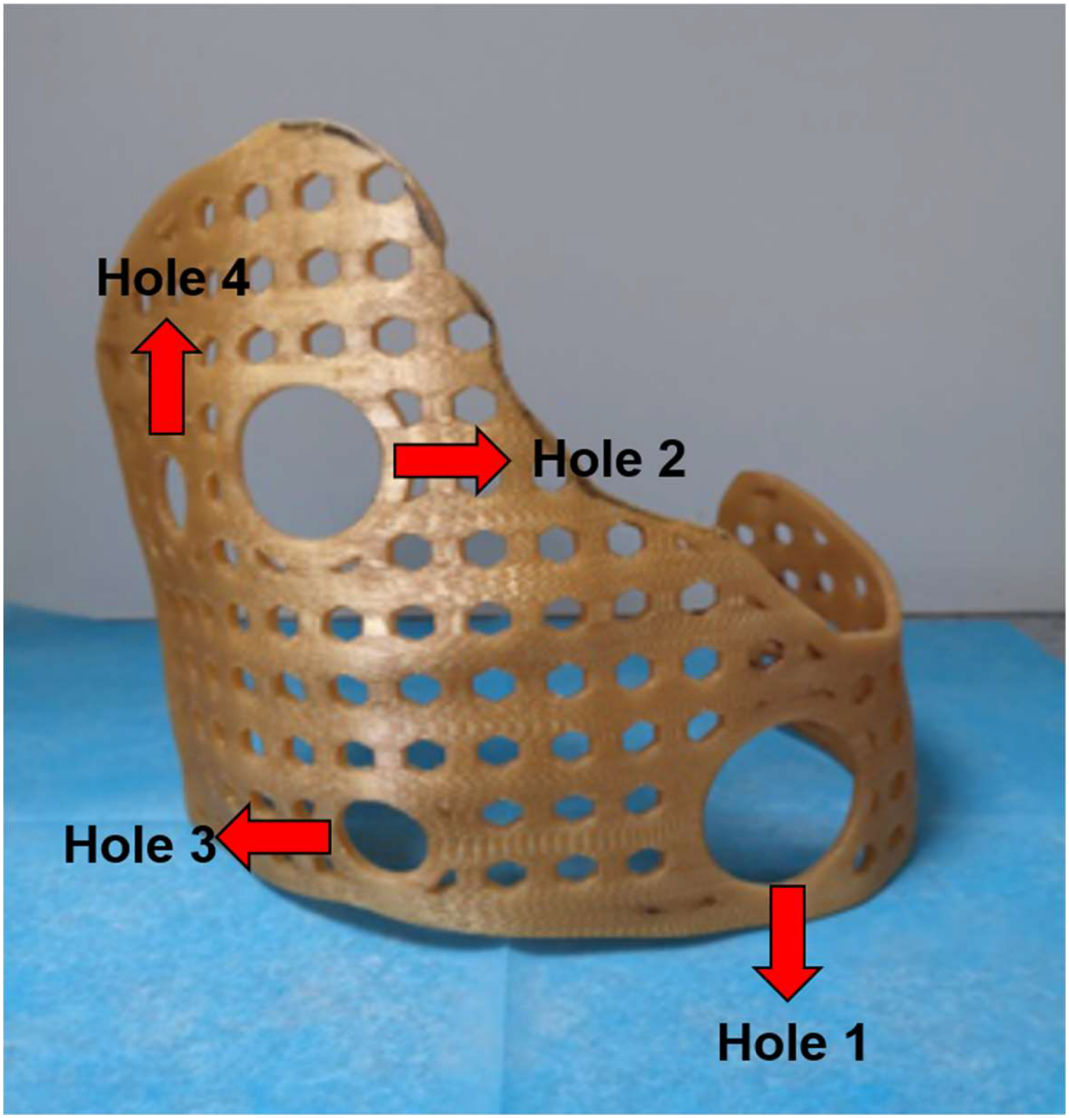
Four holes could play the role of accurate positioning, and the 3D-printed guide placed in the right place. In addition, Hole 1 and Hole 2 could also serve as operating areas for thoracoscopic and surgical instruments. The remaining holes had the same size and could be passed through by steel wire to fix the 3D-printed guide.

Intravenous anesthesia was administered by placing the patients in a lateral decubitus position with the injured side facing upward. First, the injured area of the patient was marked and the position of the external fixation guide plate was confirmed. There were four holes on the surface of the guide plate, including two large holes (Hole 1 and Hole 2) and two small holes (Hole 3 and Hole 4). The guide plate could be accurately positioned by combining the positions of the four holes. In addition to positioning, Hole 1 and Hole 2 could also serve as operating areas for thoracoscopic and surgical instruments. The combination of the two holes can be used to explore the surgical field and perform minimally invasive procedures (Figure 2).

### 2.4. Key points of 3D-printed external fixation guide

**Figure 3:**
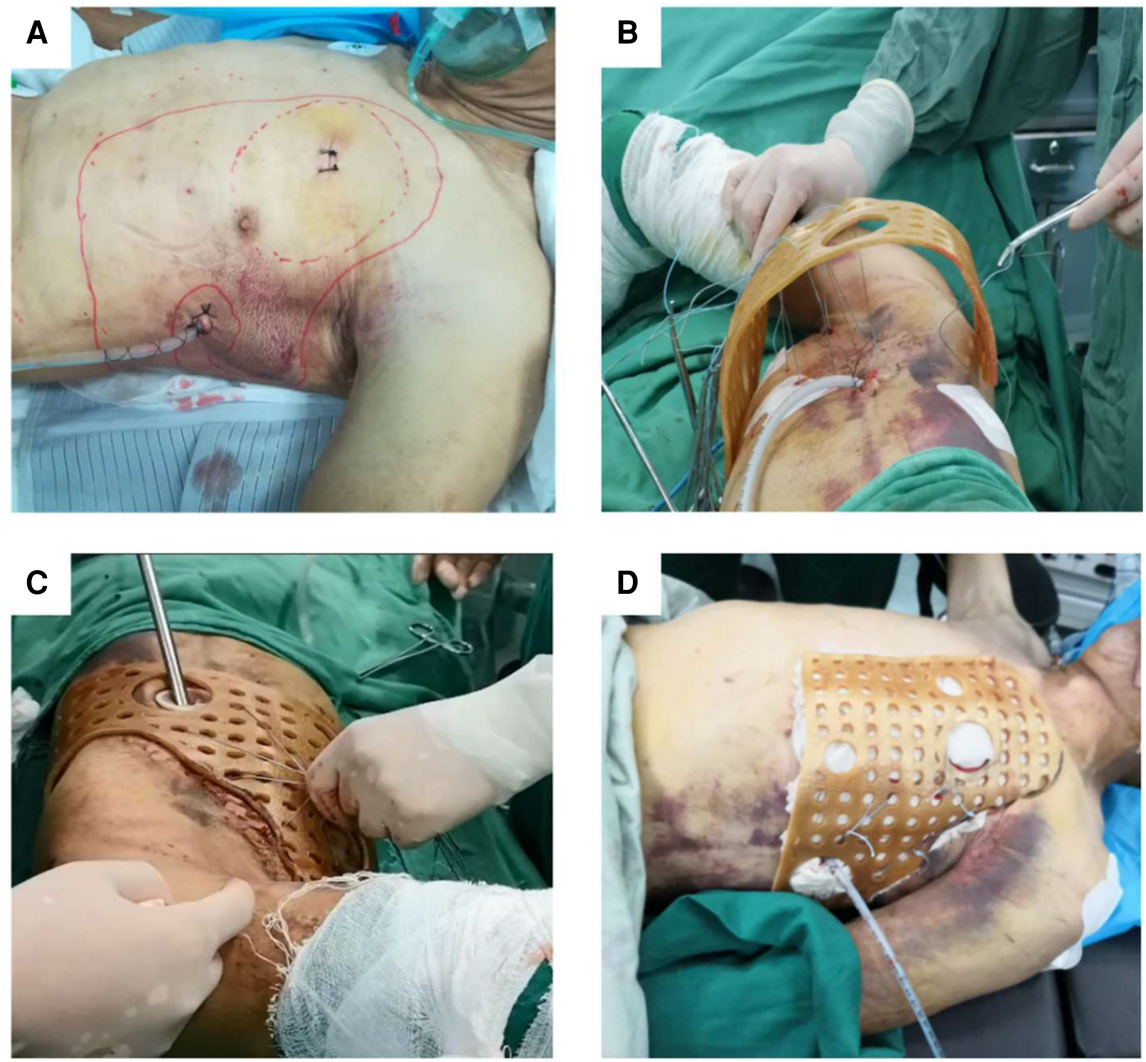
Thoracoscopy was used to explore the surgical area while adequate hemostasis was performed, the blood clot was removed, and a drainage tube was placed. (**A**) The position of the guide was initially determined by passing the steel wire through the rib fracture area and the small holes on the surface of the 3D-printed guide. (**B**) Thoracoscopic exploration was performed again to ensure that the wire passed through the rib fracture area and the collapsed ribs could be restored. (**C**) The steel wires were tightened so that the 3D-printed guide was fully fitted to the chest wall of the injured side (**D**).

After disinfecting the surgical area, the guide plate was covered in the injured area, and the site of penetration of the steel wire was confirmed after repeated testing. Then, the guide plate was pre-fixed; the assistant held the steel wire and placed the guide in the set position. In the first step, the thoracoscope was inserted through Hole 1, and thoracic exploration was performed to observe the specific conditions in the thoracic cavity, stop bleeding, and remove blood clots. In the second step, the steel wire was fixed. Under the supervision of the thoracoscope, the steel wire was fixed in the injured area to avoid damage to the blood vessels and nerves. In the third step, the 3D-printed guide was fixed. After repeated comparison of the thoracoscopic visual field and actual guiding position and confirmation, the wire on the surface of the guide plate was tightened. In the fourth step, we tested whether the guide plate was firm. To achieve the goal of effective fixation, the assistant pushed and pulled the fixed guide repeatedly to confirm that the guide was placed in the injured area (Figure 3).

For overlapping rib fractures, we confirmed the specific location and severity of rib fractures through the thoracoscope. First, we passed the steel wire through the intercostal space and wrapped it around the rib to perform local traction outside the chest wall surface. Orthopedic instruments were simultaneously used to support the collapsed fracture from within the chest cavity, and both internal and external forces were concurrently applied to achieve the goal of rib fracture reduction. Then, the steel wire was quickly fixed on the 3D printing guide plate. For those with sharp rib ends, an ultrasound bone knife was used to polish the broken ends flat under thoracoscopy during surgery to avoid puncturing adjacent tissues and causing bleeding or pain. Distinct from other limbs and trunk bones, ribs do not need to support the body's weight and movement shear force, and their biological stress is not significant. Simple external fixation can maintain thoracic stability and correct abnormal breathing. A monofilament stainless steel 316 L suture with a 48-mm diamond point needle was used for surgery (Covidien™, USA) (Figure 3). During the surgery, a steel wire with a needle was used to pass through the rib gap, and the steel wire was wrapped around the ribs for traction and fixation (Figure 3). When removing the steel wire, one side of the wire was tightly cut against the skin and pulled out (similar to the suture removal process).

## 3. Results

### 3.1. Demographic data of selected cases

From January 2020 to December 2022, five patients [4 males (80%) and 1 female (20%); age: 23–71 years, average age: 45.7 years] with multiple rib fractures and flail chests who met the inclusion criteria were enrolled (Table 1). The average number of rib fractures was 8.4 and the location of rib fractures includes the anterior, median and posterior arch (Table 2); all patients had lung contusion, hemopneumothorax, and anomalous respiration and were admitted to the intensive care unit (ICU) for monitoring. Closed thoracic drainage was performed to relieve atelectasis, and to ensure airway patency, endotracheal intubation was performed. Among the five patients, three had traffic injuries (60%) and two had impact injuries (40%), with an average preoperative visual analog scale (VAS) score of 7.0. The cost of 3D modeling and design for each patient was $204.11, and the cost of 3D printing of an external fixation guide was $476.25-$680.36. The average cost per person was approximately $571.50 (Table 3).

**Table 1:** Demographic data of patients.

Variables	Sample (*n* = 5)
Average age (years)	45.7
Sex, *n* (%)	
Male	4 (80%)
Female	1 (20%)
The average number of rib fractures	8.4
Average VAS score (pre-operation)	7
Anomalous respiration, *n* (%)	5 (100%)
Hemopneumothorax, *n* (%)	5 (100%)
Cause of injury, *n* (%)	
Traffic injury	3 (60%)
Impact injuries	2 (40%)
Pulmonary contusion, *n* (%)	5 (100%)
Closed thoracic drainage, *n* (%)	5 (100%)
Admitted to the ICU, *n* (%)	5 (100%)
Endotracheal intubation, *n* (%)	5 (100%)

**Table 2:** Number and location of rib fractures in flail chest patients.

Patient number	Anterior arch	Median arch	Posterior arch	Totality
1	3	3	3	9
2	2	3	3	8
3	4	3	3	10
4	3	2	3	8
5	1	3	3	7

**Table 3:** Clinical index.

Outcomes	Case 1	Case 2	Case 3	Case 4	Case 5	Mean value
Operation time (min)	45	30	39	25	42	36.2
Blood loss (ml)	50	15	25	36	34	32
Drainage time (day)	6	3	3	4	6	4.4
Length of stay in ICU (day)	1	1	1	1	1	1
Cost of 3D prosthesis ($)	612.32	544.29	680.36	544.29	476.25	571.50
Removal time of 3D-printed guide (day)	31	28	28	28	31	29.2
Thoracic volume recovery rate (%)	98.03	76.15	83.79	77.32	90.16	85.09
Preoperative VAS score	8	6	9	5	7	7
Postoperative VAS score	1	1	1	2	1	1.2

### 3.2. The 3D-printed guide material

The patient's waiting time for surgery is 10–24 h because of the following reasons: (1) The production of the external fixation guide plate for the chest is divided into three stages: 3D modeling, 3D printing, and post-processing. Due to the need for doctor-engineer interaction in 3D modeling, it takes at least 2–4 h to complete the modeling process. The time for 3D printing is comparatively constant, and after receiving the digital model, the product printing can generally be completed within 6 h. The repair and polishing of the external fixation guide plate can be done in the later stage and completed within 1 h; (2) If the city has a complete 3D printing medical device system, 3D printing products can be obtained within 24 h, or else, it may take 2–3 days; (3) We are an expert unit of the 3D Printing Medical Device Professional Committee of the China 3D Printing Medical Device Industry Association; therefore, the time from patient admission to obtaining 3D printed products can be as short as 10 h and no longer than 24 h. For patients with severe respiratory insufficiency caused by abnormal breathing, positive pressure ventilation with a ventilator can be first used for internal fixation, while waiting for the external fixation guide plate. For patients with progressive hemothorax or suspected damage to vital chest organs, emergency surgery is necessary for internal fixation and they cannot wait for the production of external fixation guides.

**Figure 4:**
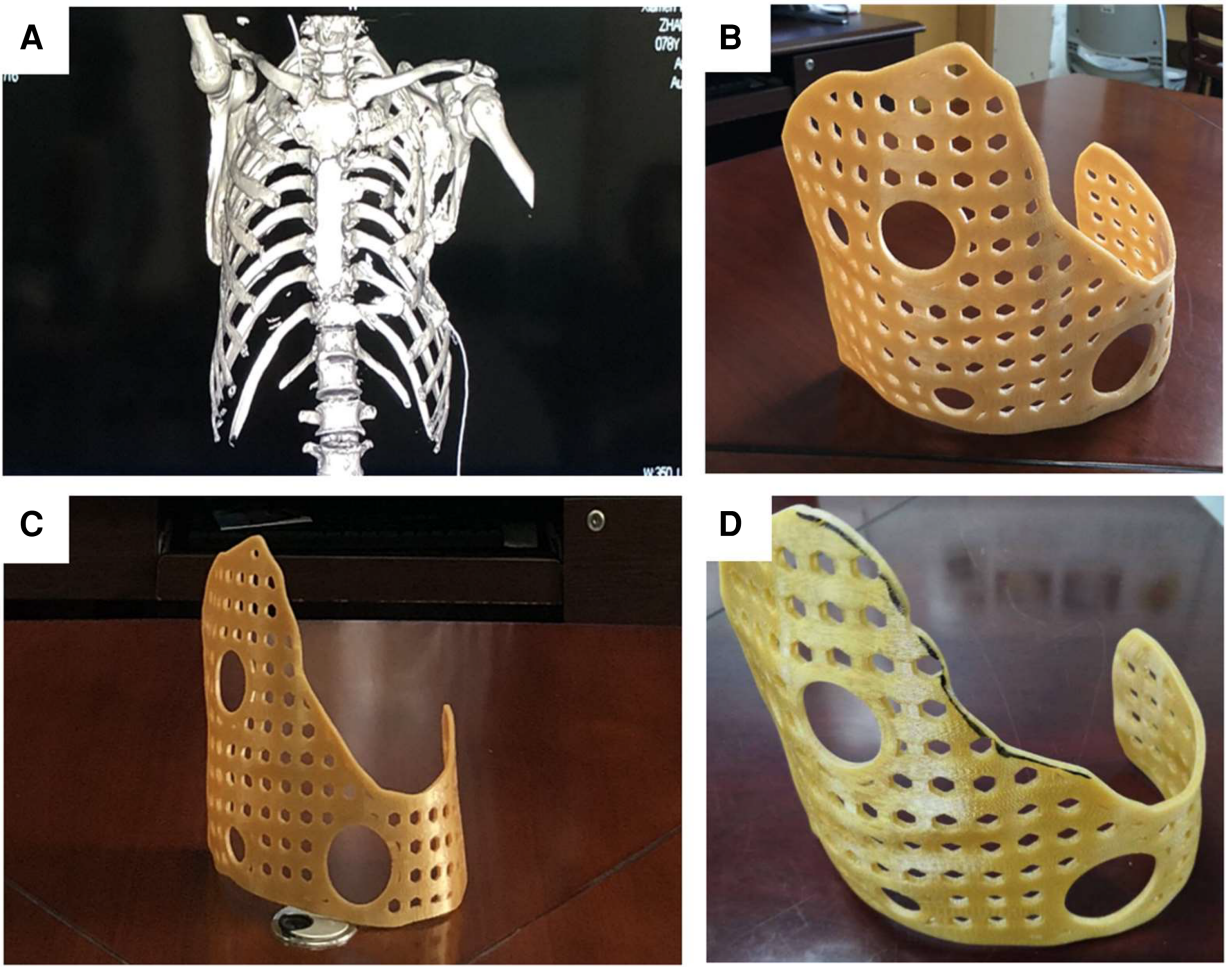
The model data designed by MIMICS was imported into the laser 3D printer to produce the external fixed guide. (**A**) The 3D-printed guide could surround the thorax, and its surface was designed with positioning and fixed holes for the wire to pass through. The guide had a larger front surface and a smaller rear surface, which could achieve the purpose of wearing comfortably and effectively wrapping (**B,C**). According to the actual situation of the patient, it was modified and polished, and finally, the 3D-printed guide for surgery was obtained (**D**).

The guide data was imported into the 3D printer and printed with strong, environmentally friendly, and easy-to-shape materials. Finally, a real guide could be obtained. According to the actual situation of the patient's chest wall, the shape of the guide was adjusted and polished repeatedly to ensure that the contact area was sufficient and firmly fixed, while not affecting the patient's daily functional exercise. After debugging, disinfect and set aside (Figure 4).

### 3.3. Parameter selection and outcomes

**Figure 5:**
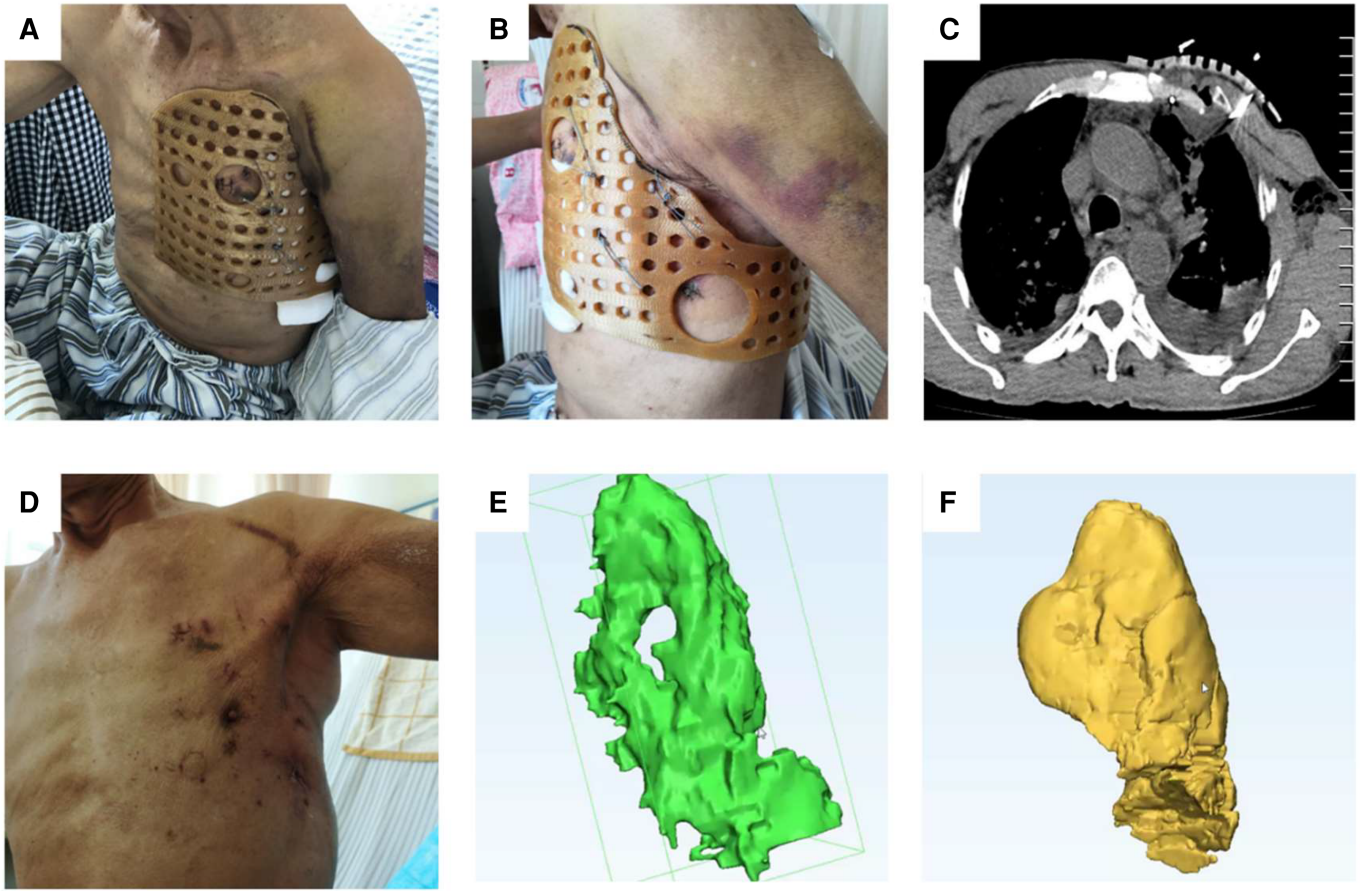
After the operation, the 3D-printed guide plate was firmly and closely fitted to the chest wall from different visual fields (**A,B**). Reexamination of the chest CT showed that the broken end of the rib fracture was reduced and the shape of the chest was reconstructed (**C,D**). Modeling was performed using MIMICS software, and the green area represents the preoperative thoracic volume (**E**) The yellow area represents the postoperative thoracic volume, which increased significantly compared to the preoperative one (**F**).

The surgical technique was assessed from the following five aspects: surgical, rehabilitation, functional, special, and follow-up parameters. The surgical parameters included operating time and blood loss. Rehabilitation parameters include drainage time, length of stay in ICU, and removal time of 3D-printed guide. Special parameters are the most important part of all indicators. Modeling was performed using CT data to evaluate the recovery of thoracic morphology and thoracic volume before and after surgery. The green area represents the volume of the thoracic cavity damaged after injury, and the yellow area represents the volume of the thoracic cavity reconstructed using the 3D-printed external fixation guide. The efficiency of the surgical technique was evaluated by mathematical modeling and comparison of the volume differences between the two color areas (Figure 5). The functional parameters included preoperative and postoperative scores, and we selected visual analog scores (VAS). The overall score was 10, with higher scores indicating more severe pain and lower scores indicating less pain. Patients chose their scores according to their actual situation (Table 3). The removal of steel wires in these five patients was smooth, with no bleeding or other injuries.

### 3.4. Follow-up information on patients

The following indicators were chosen to evaluate the postoperative status of the patients. Chronic pain is defined as pain lasting for more than 1 month. Paradoxical respiration is a pathological respiratory movement, wherein the chest wall descends during inspiration and rises during expiration, which is caused by multiple rib fractures in the chest following chest trauma and softening of the chest wall due to the loss of complete rib support. Thoracic deformity refers to the condition that the shape of the chest wall is inconsistent with that of the healthy side. Superficial wound infection, limited to the skin and subcutaneous tissue involved by the incision, occurs within 30 days after surgery. Hospital readmission refers to patients returning to the hospital for continued treatment due to flail chest and associated complications. Return to daily life means no difference in the ability to complete tasks compared with the pre-injury condition (Table 4).

**Table 4:** Clinical outcomes.

Indicators	Number (%)
Chronic pain	1 (20%)
Paradoxical respiration	0 (0%)
Thoracic deformity	0 (0%)
Superficial wound infection	1 (20%)
Hospital readmission	0 (0%)
Return to daily life	5 (100%)
Lost to follow-up	0 (0%)
Death	0 (0%)

The results of surgical parameters showed that all five patients underwent surgery successfully, and the operative time was less than 1 h (mean value: 36.2 min). Operative time was defined as the time from skin incision to wound closure. Compared with traditional ORIF, the operation time is significantly shortened (12). Blood loss was estimated based on the volume of blood in the negative pressure aspirator and the number of gauze pieces used. The intraoperative blood loss was 32 ml, which had little impact on the patient's circulatory system. Regarding rehabilitation parameters, all patients were transferred to the ICU for postoperative monitoring (mean value: 1 day), during which symptomatic support treatment was provided; the patients were moved to the general ward after their vital signs stabilized. To prevent pneumothorax and eliminate pleural effusion, an indwelling drainage tube was inserted and negative pressure aspiration was performed in all patients. The drainage time was the duration from the end of the surgery to the removal of the drainage tube. The drainage tube could be removed in an average of 4.4 days, which reduced the discomfort of patients and was beneficial to their daily activities.

The patients could get out of bed and move around 6 h postoperatively, the chest pain was significantly reduced, and the cough function was greatly restored. The patient's rotation was not affected, but when lying on the injured side, there may be obvious discomfort or pain due to compression on the external fixation guide plate. To avoid displacement of the external fixation guide, we recommended the patients avoid lying on the injured side.

Special parameters could precisely reflect the surgical effect and intuitively evaluate the postoperative condition of patients. The surgical effect is mainly based on three indicators: recovery of chest volume, correction of abnormal breathing, and improvement of pain. Due to the interference of many factors in the measurement of lung function in post-traumatic patients, the values are frequently inaccurate, and preoperative patients are usually in a post-traumatic emergency state and they were unable to cooperate with lung function measurement. Therefore, we did not perform lung function measurements. Approximately 1 month postoperatively (mean value: 29.2 days), all patients went to the outpatient clinic for a re-examination of chest CT, and the evaluation results were as follows: good rib healing, callus formation, and fuzzy fracture lines. At this time, the 3D-printed external fixation guide could be removed. By comparing preoperative and postoperative CT data, Mimics software was used to reconstruct the thoracic cavity. The rib and lung structures were removed from the model, and the cavity obtained was the thoracic volume. The preoperative thoracic volume was defined as A, the postoperative thoracic volume as B, and the recovery rate of thoracic volume was (B−A)/A. The thoracic volume recovered well postoperatively and was significantly higher than that preoperatively. The results showed that the thoracic volume recovery rate was approximately 90% (mean value: 85.09%), atelectasis was solved, and restrictive ventilation dysfunction was corrected.

For functional parameters, the postoperative VAS score (1 month postoperatively) was significantly lower than the preoperative score, and the pain symptoms of patients were significantly relieved, which helped patients to continue rehabilitation exercises.

Patients were followed up systematically 6 months after surgery. No complications such as paradoxical respiration and thoracic deformity were seen in the five patients (Table 4). Only one patient had symptoms of chronic pain postoperatively, and one patient had poor wound healing and superficial infection due to early incorrect bathing. No patient returned to the hospital for treatment, lost follow-up, or died. All patients were able to complete the tasks of daily life independently, and their quality of life was significantly improved compared with that before the operation.

## 4. Discussion

Damage control surgery (DCS) means simple surgery performed early in case of trauma can save critically ill patients who were previously thought to be impossible to treat (13, 14). DCS has recently gradually shifted the focus from abdominal trauma to cardiothoracic surgery and has made great progress, significantly reducing the mortality of patients with severe chest trauma (15, 16). According to DCS and pathophysiology, multiple rib fractures do not need to be completely fixed; only the important part needs to be fixed to restore the stability of the thorax, maintain normal physiological function, effectively relieve chest pain, and facilitate accelerated rehabilitation (17, 18). Flail chest can lead to abnormal breathing and progressive hemothorax, seriously threatening the patient's life (19). Due to the particularity of the condition, flail chest patients often need to stabilize the chest wall, stop the broken ribs from moving with breathing, restore the effective volume of the chest, and ensure normal lung ventilation (20, 21). Traditional surgical methods often use ORIF, including steel plates and screws to reconstruct the shape of fractured ribs (17, 20, 22). However, this method has the disadvantages of longer hospital stays and slower recovery (23–25). Moreover, this technique requires a secondary surgery to remove the internal fixation, further increasing the surgical trauma and cost (26–28). Therefore, discovering a surgical method with less trauma and cost that can achieve the goal of rapid recovery has become the focus of attention of thoracic surgeons. The use of minimally invasive techniques to treat flail chests is still being explored; it may become popular in the future (29). The 3D-printed external fixation guide used in the present study avoids incision and placement of internal fixation done in traditional surgery and uses a simpler external fixation technology instead. Through VATS, the internal and external visual fields were combined, which could also achieve good reduction and fixation of flail chest.

The 3D-printed external fixation guide was fixed to the outer surface of the chest wall via a steel wire. Combined with the tension of the steel wire and the strain of the guide, the contact area between the flail chest region and the external fixator was increased, to achieve the purpose of effectively supporting the fracture. When combined with VATS, the reduction of rib fractures can be assessed under direct vision by observing the medial side of the chest wall. Furthermore, intrathoracic hemostasis and removal of hemorrhage can efficiently decrease the incidence of atelectasis and pulmonary infection. The surface wounds are only thoracoscopy, observation, and tiny wire entry and exit holes, which are more minimally invasive than traditional incision surgery wounds. Each external fixation guide plate is customized according to the patient's CT data, which can better meet the actual needs of the disease and avoid individual differences affecting the treatment effect. The 3D-printed guide will be “tried on” before surgery. On the premise of ensuring the full coverage of the abnormal respiratory area, the excess part of the guide was trimmed, especially the underarm part was cut to avoid compression of the axillary nerve. Sand the cut area to make it smooth and avoid breaking the skin. During intraoperative procedures, gauze patches are placed on the edge of the guide plate to further protect the surrounding skin. At present, the 3D-printed external fixation guide involved in this study is the first in the world for the treatment of flail chest, and no relevant reports have been retrieved in PubMed, so this study is original and the research results are persuasive.

From the surgeon's perspective, this technique is simple to operate, as long as the doctor who has preliminarily mastered the use of thoracoscopy performs the surgery. The core of this new technique includes two parts, namely thoracoscopic exploration and external fixation of a 3D-printed guide. Installation of the guide plate is comparatively simple, providing the guide completely covers the area of abnormal breathing, it can be installed according to the preoperative calibration range; the external fixation can be completed with the wire and needle under thoracoscopic monitoring. Moreover, thoracoscopic exploration is a basic surgery for thoracic surgeons. The technology is simple to implement, has a short learning curve, and can be advanced with simple training. Therefore, junior doctors can also independently operate. The new surgical technique reduces surgical time, accelerates recovery time, and avoids secondary surgical fixation. Although no comparative study has been conducted, this technique still has obvious advantages over previous techniques and can significantly reduce medical expenses. At present, a rib internal fixation plate with a length of 15 cm costs $3,429.35 per piece and the internal fixation screw costs $205.76 per piece. To complete the internal fixation of a flail chest plate, the material cost is usually approximately $6,858, and the medical cost for secondary surgery to remove the plate is also approximately $4,115, which is similar to the cost in Brazil (30). In comparison, the overall cost of the 3D thoracic external fixation guide plate does not exceed $680.36, which has a huge economic advantage.

All the patients included in the present study had high-energy trauma, and the causes were traffic accidents and violence. The higher VAS score before surgery indicated that the pain symptoms were obvious, and the postoperative score was significantly reduced, which proved that the new technology alleviated the discomfort of patients. Early pain control can promote patients to do early rehabilitation exercises and reduce the incidence of lung infection, urinary tract infection, and deep venous thrombosis of lower limbs (31, 32). The operative time of 5 patients with flail chest was short, and the external fixation of a 3D-printed guide with steel wire and adjustment of guide position accounted for most of the time. These operations were carried out on the body's surface without increasing the trauma and blood loss of patients. Most of studies on multiple rib fractures have focused on 3D printing assisted internal fixation (rib locking plate was pre-shaped), with surgery times mostly around 120 min (Supplementary Table S1). Zhou et al. reported that the intraoperative blood loss was 100–150 ml in 3D printing assisted internal fixation (33). By contrast, the 3D-printed guide technology has less operation time and intraoperative bleeding, only approximately 36.2 min and 30 ml, respectively; thus, the surgery has little impact on the hemodynamics of patients, avoiding postoperative blood transfusion and prolonged ICU monitoring. The 3D-printed guide fixation under thoracoscopic supervision can achieve a near-anatomical reduction of rib fractures. After the reconstruction of the thoracic contour, the thoracic volume is restored and restrictive ventilation dysfunction is avoided. In the present study, the thoracic volume recovery rate was 85.09% on average and 98.03% on the highest, which was equivalent to the thoracic volume of the uninjured side. Solid external fixation can prevent abnormal breathing; coordinated respiratory movements can decrease the occurrence of adverse events such as respiratory failure and lung injury.

While having the 3D-printed external fixation guide, patients can easily perform early activities without long-term bed rest, which decreases the occurrence of deep vein thrombosis, hypostatic pneumonia, and pressure soreness. Early thoracic support can support the formation and shaping of primitive callus (32). During this period, as the soft tissue edema subsides, we can refix the guide by adjusting the tightness of the steel wire as required to ensure that the guide closely fits the chest wall soft tissue. In the present study, the average time of wearing the external fixation guide plate for the patients was only approximately 30 days. Compared with the traditional internal fixation surgery, the rehabilitation time with this new technique was significantly shortened, and there was no requirement for secondary surgery after removing the guide, which reduced the surgical trauma and cost (34).

However, few postoperative complications were noted during the follow-up with the use of this method. One patient developed chronic pain, which lasted for 35 days (VAS score: 2). After posterior intercostal nerve block treatment, the pain symptoms disappeared. There was also an obese patient with wound fat liquefaction, which slowly healed and developed a superficial wound infection. The wound healed well following continuous wound dressing changes and re-sutured.

The results of this study showed that a 3D-printed external fixed guide combined with VATS has obvious advantages in the treatment of flail chest, but there are still some limitations. First, the sample size of this study was small. With the improvement of safety awareness, patients with flail chest are becoming rarer. Only five patients were enrolled in this study based on the inclusion criteria, which is not enough to show that this technique was suitable for the vast majority of patients with flail chest. In future research, multicenter collaboration is needed to increase the sample size, so that the results can be more convincing. Furthermore, the short follow-up time of this study only shows that the short-term clinical effect of 3D printing technology combined with VATS of flail chest is satisfactory; however, the long-term effect needs further observation. Short follow-up time covers the occurrence of some complications and cannot fully assess the efficacy of this surgical technique. Moreover, the widespread application of this surgical method is limited because 3D printing technology has not yet been fully popularized. This study is a case series with limited convincibility and credibility. With the increase of sample size in the future, the next step is to design a randomized controlled study to compare this technique with traditional ORIF surgery and further verify the advantages of 3D printing technology in the treatment of flail chest.

## 5. Conclusions

This is the first clinical evidence with a series of cases of flail chest treated with a 3D-printed external fixed guide combined with VATS. The procedure is safe, effective, feasible, and minimally invasive and has great potential and application prospects in clinic settings.

## Data Availability

The raw data supporting the conclusions of this article will be made available by the authors, without undue reservation.

## References

[1] de Campos JRM White TW. Chest wall stabilization in trauma patients: why, when, and how? J Thorac Dis. (2018) 10(Suppl 8):S951–S62. 10.21037/jtd.2018.04.69 29744222 PMC5934118

[2] Ma X Dong Z Wang Y Gu P Fang J Gao S. Risk factors analysis of thoracic trauma complicated with acute respiratory distress syndrome and observation of curative effect of lung-protective ventilation. Front Surg. (2021) 8:826682. 10.3389/fsurg.2021.826682 35141272 PMC8818796

[3] Hoepelman RJ Minervini F Beeres FJP van Wageningen B IJpma FF van Veelen NM Quality of life and clinical outcomes of operatively treated patients with flail chest injuries: a multicentre prospective cohort study. Front Surg. (2023) 10:1156489. 10.3389/fsurg.2023.1156489 37009603 PMC10050428

[4] Balogh ZJ. Rib fracture fixation: where and what is the baseline? Injury. (2021) 52(6):1239–40. 10.1016/j.injury.2021.05.021 34051981

[5] Aparicio-Blanco BS Rio RB Cabrera LF Sanchez-Ussa S Martinez S Serna-Lozano A Early fixation of the flail chest: case report. Cir Cir. (2020) 88(Suppl 1):63–7. 10.24875/CIRU.20001505 32963408

[6] Richman AP Brahmbhatt TS Litle VR. Let's not fail the flail chest. Ann Thorac Surg. (2022) 113(6):1865–6. 10.1016/j.athoracsur.2021.06.030 34270969

[7] Beks RB de Jong MB Houwert RM Sweet AAR De Bruin I Govaert GAM Long-term follow-up after rib fixation for flail chest and multiple rib fractures. Eur J Trauma Emerg Surg. (2019) 45(4):645–54. 10.1007/s00068-018-1009-5 30229337 PMC6689022

[8] Tejo-Otero A Buj-Corral I Fenollosa-Artes F. 3d Printing in medicine for preoperative surgical planning: a review. Ann Biomed Eng. (2020) 48(2):536–55. 10.1007/s10439-019-02411-0 31741226

[9] Sun Z. 3d Printing in medicine: current applications and future directions. Quant Imaging Med Surg. (2018) 8(11):1069–77. 10.21037/qims.2018.12.06 30701160 PMC6328380

[10] Mishra S. Application of 3d printing in medicine. Indian Heart J. (2016) 68(1):108–9. 10.1016/j.ihj.2016.01.009 26896278 PMC4759482

[11] Czyżewski W Jachimczyk J Hoffman Z Szymoniuk M Litak J Maciejewski M Low-cost cranioplasty-a systematic review of 3d printing in medicine. Materials (Basel). (2022) 15(14):4731. 10.3390/ma15144731 35888198 PMC9315853

[12] Tanaka H Yukioka T Yamaguti Y Shimizu S Goto H Matsuda H Surgical stabilization of internal pneumatic stabilization? A prospective randomized study of management of severe flail chest patients. J Trauma. (2002) 52(4):727–32; discussion 32. 10.1097/00005373-200204000-00020 11956391

[13] Daskal Y Paran M Korin A Soukhovolsky V Kessel B. Multiple rib fractures: does flail chest matter? Emer Med J. (2021) 38(7):496–500. 10.1136/emermed-2020-210999 33986019

[14] Yahn CA McNally AP Deivert K Fraga T Sharaf Alddin RA Martyak MT Outcomes of trauma patients with flail chest and surgical rib stabilization. Am Surg. (2022) 88(4):810–2. 10.1177/00031348211056260 34806413

[15] Goncalves R Saad R Jr. Thoracic damage control surgery. Rev Col Bras Cir. (2016) 43(5):374–81. 10.1590/0100-69912016005017 27982332

[16] Molnar TF. Thoracic damage control Surgery. J Thorac Dis. (2019) 11(Suppl 2):S158–S66. 10.21037/jtd.2018.11.32 30906580 PMC6389564

[17] Lodhia JV Konstantinidis K Papagiannopoulos K. Surgical management of multiple rib fractures/flail chest. J Thorac Dis. (2019) 11(4):1668–75. 10.21037/jtd.2019.03.54 31179112 PMC6531730

[18] Bhatnagar A Mayberry J Nirula R. Rib fracture fixation for flail chest: what is the benefit? J Am Coll Surg. (2012) 215(2):201–5. 10.1016/j.jamcollsurg.2012.02.023 22560319

[19] Vana PG Neubauer DC Luchette FA. Contemporary management of flail chest. Am Surg. (2014) 80(6):527–35. 10.1177/000313481408000613 24887787

[20] Sawyer E Wullschleger M Muller N Muller M. Surgical rib fixation of multiple rib fractures and flail chest: a systematic review and meta-analysis. J Surg Res. (2022) 276:221–34. 10.1016/j.jss.2022.02.055 35390577

[21] Otaka S Aso S Matsui H Fushimi K Yasunaga H. Effectiveness of surgical fixation for rib fractures in relation to its timing: a retrospective Japanese nationwide study. Eur J Trauma Emerg Surg. (2022) 48(2):1501–8. 10.1007/s00068-020-01548-1 33210171 PMC7673683

[22] Owattanapanich N Lewis MR Benjamin ER Jakob DA Demetriades D. Surgical rib fixation in isolated flail chest improves survival. Ann Thorac Surg. (2022) 113(6):1859–65. 10.1016/j.athoracsur.2021.05.085 34214544

[23] Davignon K Kwo J Bigatello LM. Pathophysiology and management of the flail chest. Minerva Anestesiol. (2004) 70(4):193–9. 15173695

[24] Dogrul BN Kiliccalan I Asci ES Peker SC. Blunt trauma related chest wall and pulmonary injuries: an overview. Chin J Traumatol. (2020) 23(3):125–38. 10.1016/j.cjtee.2020.04.003 32417043 PMC7296362

[25] Deng H Tang TX Yao Y Zhang C Wu H Li ZW The incidence, clinical characteristics, and outcome of polytrauma patients with the combination of pulmonary contusion, flail chest and upper thoracic spinal injury. Injury. (2022) 53(3):1073–80. 10.1016/j.injury.2021.09.053 34625240

[26] Leinicke JA Elmore L Freeman BD Colditz GA. Operative management of rib fractures in the setting of flail chest: a systematic review and meta-analysis. Ann Surg. (2013) 258(6):914–21. 10.1097/SLA.0b013e3182895bb0 23511840 PMC3694995

[27] Pettiford BL Luketich JD Landreneau RJ. The management of flail chest. Thorac Surg Clin. (2007) 17(1):25–33. 10.1016/j.thorsurg.2007.02.005 17650694

[28] Choi J Mulaney B Laohavinij W Trimble R Tennakoon L Spain DA Nationwide cost-effectiveness analysis of surgical stabilization of rib fractures by flail chest status and age groups. J Trauma Acute Care Surg. (2021) 90(3):451–8. 10.1097/TA.0000000000003021 33559982

[29] Xia H Zhu D Li J Sun Z Deng L Zhu P Current status and research progress of minimally invasive surgery for flail chest. Exp Ther Med. (2020) 19(1):421–7. 10.3892/etm.2019.8264 31885692 PMC6913304

[30] Garcia DFV Mesias AVC Vieites L Mendes PMP Ripardo JPS. Case report: the use of three-dimensional biomodels for surgical planning of rib fixation. Trauma Case Rep. (2020) 26:100291. 10.1016/j.tcr.2020.100291 32123719 PMC7038006

[31] Coughlin TA Ng JW Rollins KE Forward DP Ollivere BJ. Management of rib fractures in traumatic flail chest: a meta-analysis of randomised controlled trials. Bone Joint J. (2016) 98-B(8):1119–25. 10.1302/0301-620X.98B8.37282 27482027

[32] Udekwu P Roy S McIntyre S Farrell M. Flail chest: influence on length of stay and mortality in blunt chest injury. Am Surg. (2018) 84(9):1406–9. 10.1177/000313481808400940 30268166

[33] Zhou XT Zhang DS Yang Y Zhang GL Xie ZX Chen MH Analysis of the advantages of 3d printing in the surgical treatment of multiple rib fractures: 5 cases report. J Cardiothorac Surg. (2019) 14(1):105. 10.1186/s13019-019-0930-y 31186011 PMC6560852

[34] van Gool MH van Roozendaal LM Vissers YLJ van den Broek R van Vugt R Meesters B Vats-assisted surgical stabilization of rib fractures in flail chest: 1-year follow-up of 105 cases. Gen Thorac Cardiovasc Surg. (2022) 70(11):985–92. 10.1007/s11748-022-01830-6 35657504

