# Supplementary material for: 3D-printed external fixation guide combined with video-assisted thoracoscopic surgery for the treatment of flail chest: a technical report and case series
Source: Front Surg. 2023 Sep 27;10:1272628. doi: 10.3389/fsurg.2023.1272628 (PMC10564999; doi:10.3389/fsurg.2023.1272628)
Supplement: Supplementary file 1 [file Table1.doc]

**Supplemental Table 1.** **Application of 3D printing in rib fractures**

| **Author and year** | **Type of study** | **Sample** | **Number of rib fractures** | **Treatment** | **Operating time (min)** | **ICU stay (day)** | **3D printing time (h)** |
| --- | --- | --- | --- | --- | --- | --- | --- |
| Chen et al. (2018) (1) | Cohort study | 17 | 5.18 ± 1.78 | 3D printing assisted internal fixation | 125.0 ± 33.4 | 6.44 ± 5.53 | 5.0-6.0 |
| Zhou et al. (2021) (2) | Validation Study | 16 | 4.0 | 3D printing assisted internal fixation | 120.0 (95-150) | NA | NA |
| Zhou et al. (2019) (3) | Case report | 5 | 7.20 ± 2.79 | 3D printing assisted internal fixation | 60.0-180.0 | 1 | NA |
| Zhou et al. (2023) (4) | Cohort study | 11 | 3.0 (1, 5) | 3D printing assisted internal fixation | 157.7 ± 67.0 | NA | NA |
| Song et al. (2018) (5) | Case report | 3 | 3.33 ± 0.47 | 3D printing assisted internal fixation | 120.0 | NA | 1.04 (0.75-1.55) |
| Lin et al. (2018) (6) | Case report | 1 | 4.0 | 3D printing assisted internal fixation | 130.0 | NA | 5.0 |
| Garcia et al. (2020) (7) | Case report | 1 | 6.0 | 3D printing assisted internal fixation | NA | 1.0 | 16.0 |
| Smith et al. (2018) (8) | Case report | 1 | 5.0 | 3D printing assisted internal fixation | 132.0 | NA | NA |

**References:**

1 Chen YY, Lin KH, Huang HK, Chang H, Lee SC, Huang TW. The Beneficial Application of Preoperative 3d Printing for Surgical Stabilization of Rib Fractures. *PloS one* (2018) 13(10):e0204652. doi: 10.1371/journal.pone.0204652.

2. Zhou X, Zhang D, Xie Z, Yang Y, Chen M, Liang Z, et al. Application of 3d Printing and Framework Internal Fixation Technology for High Complex Rib Fractures. *Journal of cardiothoracic surgery* (2021) 16(1):5. doi: 10.1186/s13019-020-01377-8.

3. Zhou XT, Zhang DS, Yang Y, Zhang GL, Xie ZX, Chen MH, et al. Analysis of the Advantages of 3d Printing in the Surgical Treatment of Multiple Rib Fractures: 5 Cases Report. *Journal of cardiothoracic surgery* (2019) 14(1):105. doi: 10.1186/s13019-019-0930-y.

4. Zhou X, Zhang D, Xie Z, Yang Y, Feng L, Hou C, et al. Application of Preoperative 3d Printing in the Internal Fixation of Posterior Rib Fractures with Embracing Device: A Cohort Study. *BMC surgery* (2023) 23(1):237. doi: 10.1186/s12893-023-02128-x.

5. Song L, Zhang Q, Ning S, Xie H, LI N, Wang Y. The Utility of 3d Printing for Implant Design for Rib Fracture Fixation. *Chinese Journal of Thoracic and Cardiovascular Surgery* (2018):288-91.

6. Lin W-C, Lin K-H, Huang H-K, Huang T-W, Chen Y-Y. Preoperative Three-Dimensional Printing for Surgical Stabilization of Rib Fractures. *Journal of Medical Sciences* (2018) 38(1):46-8.

7. Garcia DFV, Mesias AVC, Vieites L, Mendes PMP, Ripardo JPS. Case Report: The Use of Three-Dimensional Biomodels for Surgical Planning of Rib Fixation. *Trauma case reports* (2020) 26:100291. doi: 10.1016/j.tcr.2020.100291.

8. Smith JA, Ho VP, Towe CW. Using 3-Dimensional Modeling to Customize Titanium Plates for Repair of Chest Wall Trauma. *Surgical innovation* (2018) 25(2):115-20. doi: 10.1177/1553350617753225.
